# Scientific workflows for bibliometrics

**DOI:** 10.1007/s11192-016-1885-6

**Published:** 2016-02-15

**Authors:** Arzu Tugce Guler, Cathelijn J. F. Waaijer, Magnus Palmblad

**Affiliations:** Center for Proteomics and Metabolomics, Leiden University Medical Center, Leiden, The Netherlands; Faculty of Social and Behavioural Sciences, Centre for Science and Technology Studies, Leiden University, Leiden, The Netherlands

**Keywords:** Bibliometrics, Scientific workflows, Taverna, R, XML, Mass spectrometry, Medicinal chemistry

## Abstract

Scientific workflows organize the assembly of specialized software into an overall data flow and are particularly well suited for multi-step analyses using different types of software tools. They are also favorable in terms of reusability, as previously designed workflows could be made publicly available through the myExperiment community and then used in other workflows. We here illustrate how scientific workflows and the Taverna workbench in particular can be used in bibliometrics. We discuss the specific capabilities of Taverna that makes this software a powerful tool in this field, such as automated data import via Web services, data extraction from XML by XPaths, and statistical analysis and visualization with R. The support of the latter is particularly relevant, as it allows integration of a number of recently developed R packages specifically for bibliometrics. Examples are used to illustrate the possibilities of Taverna in the fields of bibliometrics and scientometrics.

## Introduction

Information processing permeates the scientific enterprise, generating and organizing knowledge about nature and the universe. In the modern era, computational technology enables us to automate data handling, reducing the need for human labor in information processing. Often information is processed in several discrete steps, each building on previous ones and utilizing different tools. Manual orchestration is then frequently required to connect the processing steps and enable a continuous data flow. An alternative solution would be to define interfaces for the transition between processing layers. However, these interfaces then need to be designed specifically for each pair of steps, depending on the software tools they use, which compromises reusability. Whether the data flow is automated or manually done by the researcher, the latter still has to deal with many detailed, low-level aspects of the execution process (Gil 2009).

*Scientific workflow managers* connect processing units through data, control connections and simplify the assembly of specialized software tools into an overall data flow. They smoothly render stepwise analysis protocols in a computational environment designed for the purpose. Moreover, the implemented protocols are reusable. Existing workflows can be shared and used by other workflows, or they can be modified to solve different problems. Several general purpose scientific workflow managers are freely available, and a few more optimized for specific scientific fields (De Bruin et al. 2012). Most of these managers provide visualization tools and have a graphical user interface, e.g. KNIME (Berthold et al. 2008), Galaxy (Goecks et al. 2010) and Taverna (Oinn et al. 2004). Not surprisingly, scientific workflows are now becoming increasingly popular in data intensive fields such as astronomy and biology.

In this paper, which builds on a recent ISSI conference paper (Guler et al. 2015), we describe the use of scientific workflows in bibliometrics using the *Taverna Workbench*. Taverna Workbench is an open source scientific workflow manager, created by the myGrid project (Stevens et al. 2003), and is now being used in different fields of science. Taverna provides integration of many types of components such as communication with Web services (WSDL, SOAP etc.), data import and extraction (XPath for XML, spreadsheet import from tabular data), and data processing with Java-like Beanshell scripts or the statistical language R (Wolstencroft et al. 2013). Beanshell services allow the user to either program a small utility from scratch and towards a specific goal, or to integrate already existing software into the workflow. The R support is a particularly powerful feature of Taverna. Although R was initially developed as a language for statistical analysis, its widespread use has seen it adopted for many tasks not originally envisioned—a fate not unlike its commercial cousin, MATLAB. One such task is text mining. The R package “tm” (Feinerer et al. 2008) provides basic text mining functionality and is used by a rapidly growing number of higher-level packages, such as “RTextTools” (Jurka et al. 2014), “topicmodels” (Grün and Hornik 2011) and “wordcloud” (Fellows 2013). Similarly, there are many toolkits and frameworks for text mining in Java that could also be called from within a Taverna workflow. For geographic and geospatial analysis, e.g. using author affiliations, there are also a number of very powerful R packages. One such package is “rworldmap” (South 2011), projecting scalar, numerical data onto a current map of the world using the ISO 3166-1 country names. rworldmap gives the user control of most aspects of the map drawing, and enables different map projections to be applied to the maps.

### A simple example: comparing two authors

**Fig. 1:**
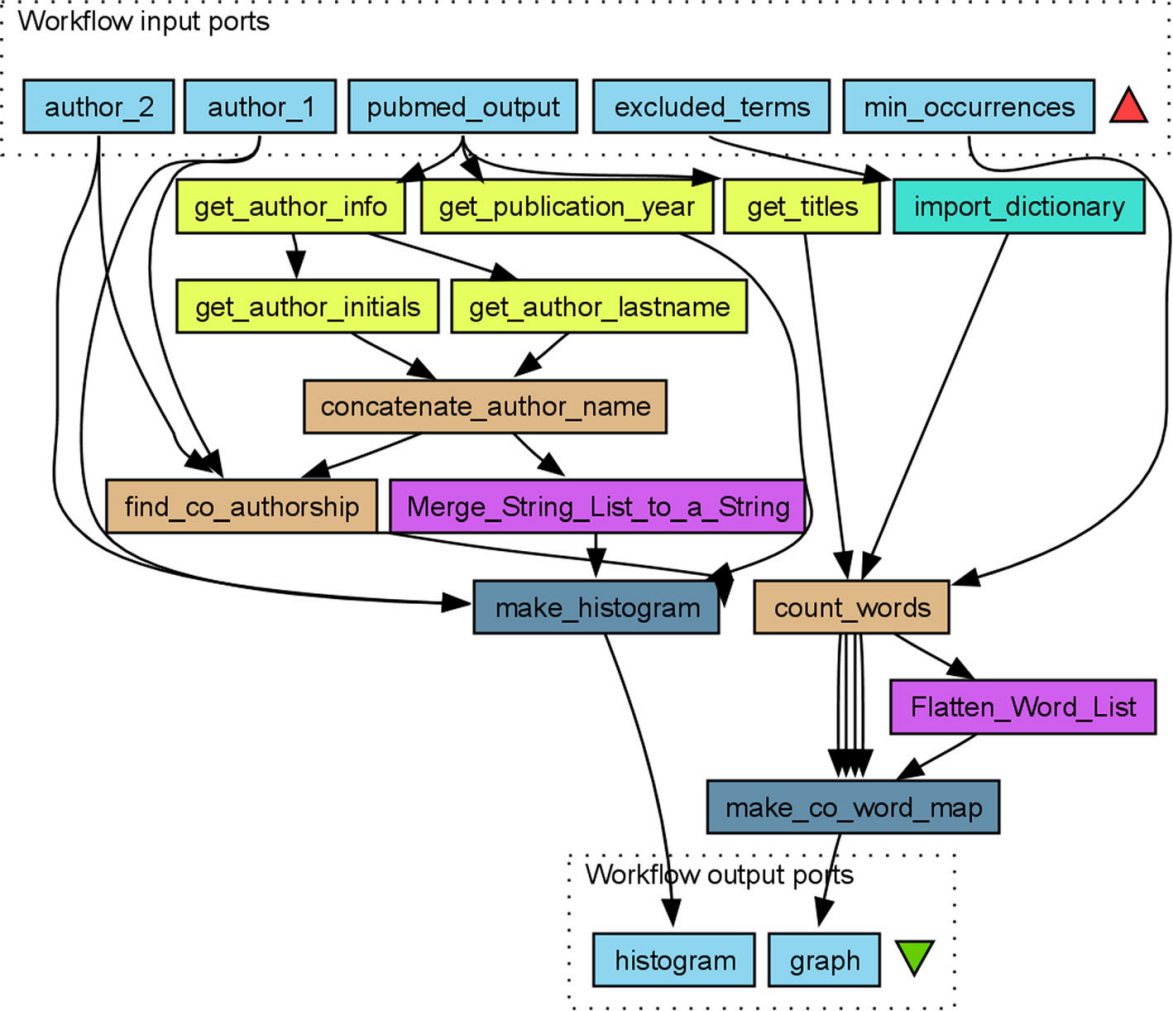
A workflow *Compare_two_authors* designed in Taverna for comparing the scientific output over time and word usages of two researchers (authors). Taverna uses *color* to indicate the type of service or tool. Although not performing a particularly sophisticated bibliometric analysis, this workflow demonstrates the use of Beanshells (*burly wood brown*), local services (*heliotrope violet*), spreadsheet import (*turquoise*), XPaths (*laser lemon yellow*) and Rshells (*air force blue*). The inputs (*sky blue*) are some PubMed results in XML, the names of two authors, a dictionary of excluded terms and the minimum number of occurrences. Each execution of the workflow creates two outputs: a histogram of the publications in each year for the two authors and a co-word map comparing their research topics. Common words can be excluded for clarity. The *import_dictionary* spreadsheet import service is used to read a text file with one word per line containing words to be excluded

We designed a simple workflow, *Compare_two_authors* (Fig. 1), to generate a histogram for the number of publications over time and a co-word map for the titles of the two authors’ publications. The workflow takes as inputs PubMed results in XML, the names of two authors, a list of excluded words and a minimum number of occurrences.

**Fig. 2:**
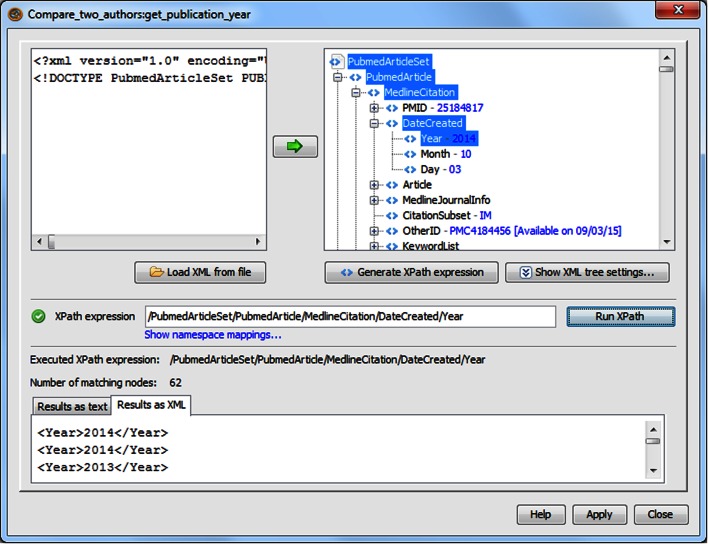
The XPath configuration pane provides a simple interface for extracting particular data fields from XML files, here publication years from PubMed search results in XML. There are several “years” in a PubMed entry, corresponding to the date-of-creation for the Medline citation, the article publication date or journal issue publication date. Only the Medline citation date is always present. The XPath/PubmedArticleSet/PubmedArticle/MedlineCitation/DateCreated/Year extracts the year from this date

The PubMed results are retrieved in an XML format, and the extraction of publication years, titles and author names are done by *XPath* services. XPath is a query language for selecting elements and attributes in an XML document. The XPath service in Taverna eases this process by providing a configuration pane to render an XML file of interest as a tree and automatically generate an XPath expression as the user selects a specific fragment from the XML (Fig. 2). The results of the query can either be passed as text or as XML to other workflow components.

The data extracted by the spreadsheet import and XPath services is fed to a series of Beanshell components that find co-authorships and count co-occurrence of words in the extracted titles. Beanshell is a light-weight scripting language that interprets Java. In our workflow, the Beanshell services do simple operations on strings, such as concatenation of surnames and initials that are extracted separately using XPath (*concatenate_author_names*), matching strings to find co-authorships (*find_co_authorship*) and counting the number of words occurring in each title authored by one or both authors (*count_words*). The two authors’ usage of the words, excluding *excluded_terms*, that appear at least *min_occurrences* times in total, are then used to draw a co-word map using the “igraph” R package (Csárdi and Nepusz 2006). Excluded terms may be very common, non-informative words like articles and prepositions that would not carry any meaning in a co-word map. It is generally up to the workflow designer what part of the workflow to code in Java (Beanshell), in R, or in third language called via the *Tool* command-line interface. More types are available for data connectors between R components (logical, numeric, integer, string, R-expression, text file and vectors of the first four types) than between Beanshell components, where everything is passed as strings. Therefore, when dealing with purely numerical data, we recommend R over Beanshells within Taverna.

**Fig. 3:**
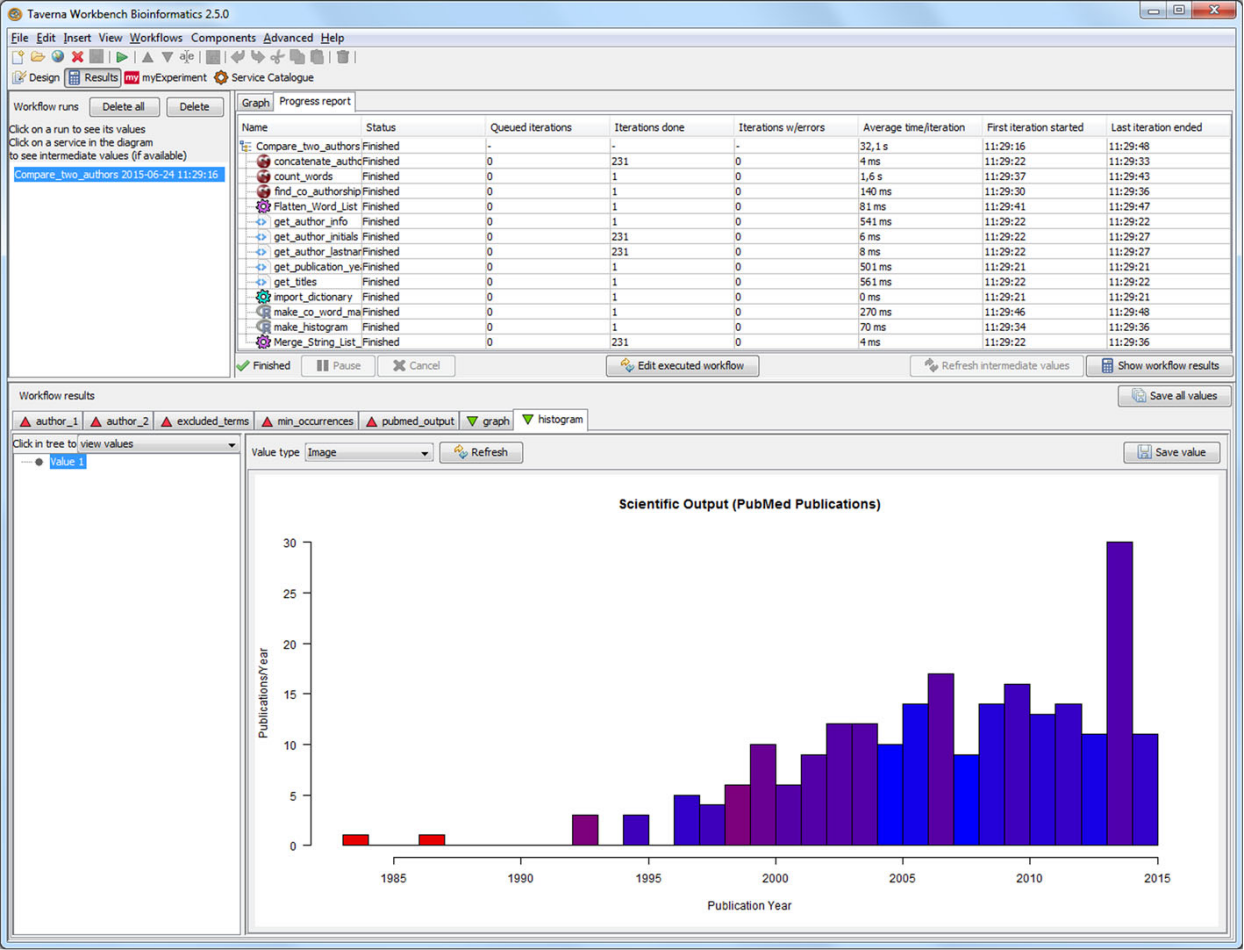
Workflow Progress report in the Taverna workbench Results perspective—here with a completed execution of the *Compare_two_authors* workflow in Fig. 1. The “histogram” output is here captured by Taverna, allowing the user to browse the results and select what to save or export to a different data format. In this particular case, the histogram is colored according to relative author output, with *red* being Glish and *blue* McLuckey

**Fig. 4:**
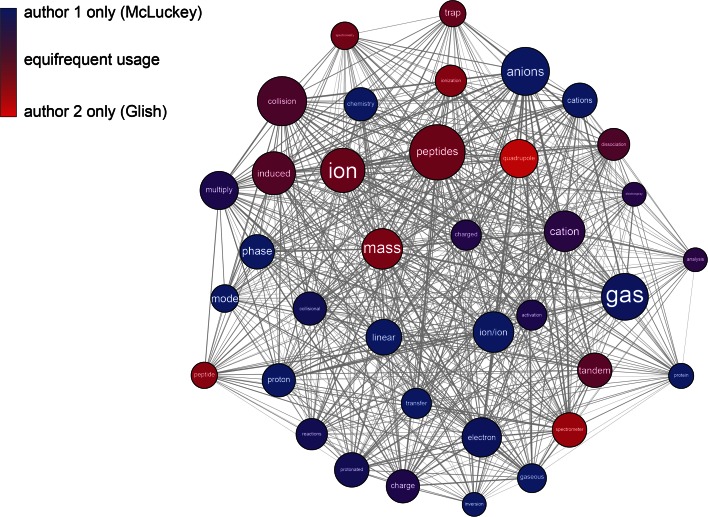
Co-word map output from the *Compare_two_authors* workflow. Graphical output from an Rshell can either be in PNG format to be captured by Taverna and browsed in the Results perspective or in a vector format such as SVG or PDF. A workflow can start from data available online, orchestrate all processing and analysis steps, and produce figures or charts in PDF, suitable for publication. Such workflows enable readers to replicate exactly what was published by another researcher with a few mouse clicks. As in the histogram in Fig. 3, *red* stands for Glish and *blue* for McLuckey

After all the necessary inputs are provided, the workflow is ready to be executed. In the Taverna Workbench Results perspective (Fig. 3), each completed process is grayed out to show the progress of the workflow run. The execution times, errors and results are also visible in this perspective. We ran the workflow for two scientists active in our own field of mass spectrometry: Gary L. Glish and Scott A. McLuckey, whom we knew to have worked on similar topics over a long period of time and also co-authored a number of articles. However, the workflow will work on any two authors with publications indexed by PubMed. The co-word map in Fig. 4 visualizes the co-occurrence of words in titles by the location and thickness of the connecting edge, while the relative frequency of usage by the two authors is indicated by color (here from red to blue). This is an example meant to illustrate the capabilities of scientific workflows, not to show a difficult or even particularly interesting bibliometric analysis, although we were surprised to see how strongly individual language preferences appear in this maps, even for two researchers who have a long history of collaboration. For example, one researcher (Glish) may have a strong preference to specify that a “quadrupole ion trap” was used in an experiment whereas another (McLuckey) may refer to the same apparatus as simply an “ion trap”.

### Citation analysis

**Fig. 5:**
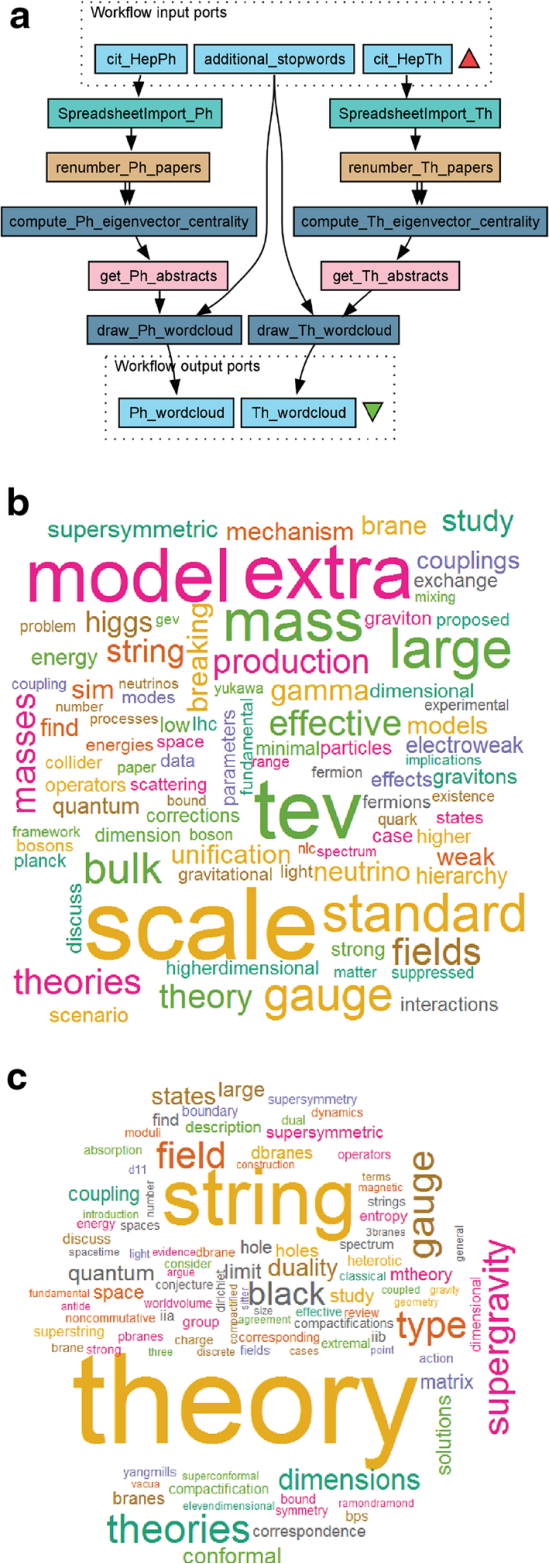
Taverna workflow for citation analysis (**a**) and wordclouds for 100 core papers in the high-energy physics phenomenology (**b**) and theory (**c**) citation networks as defined by eigenvector centrality

As a second example, we will use a Taverna workflow to analyse citation networks. Citation networks are widely used in bibliometrics to study patterns of who-cites-whom and to study associations between academic groups or areas of research. To simplify the example, we will start from existing networks and compare the citation networks cit-HepPh and cit-HepTh (Leskovec et al. 2005), which show the citation relations between all papers published in the e-print archive *arXiv* between January 1993 and April 2003 on high-energy physics phenomenology (cit-HepPh) and high-energy physics theory (cit-HepTh). Specifically, we compare the eigenvector centrality of the papers in both networks. The eigenvector centrality is a measure of the importance of a node in the network and depends on the centrality of the nodes it is linked to (Bonacich 2007). The workflow (Fig. 5a) takes as input the citation graphs as edgelist files, as available from the Stanford Large Network Dataset Collection (https://snap.stanford.edu/data/). SpreadsheetImport services are used to read the edgelist tables, skipping the first four header lines of each. The arXiv paper identifiers are renumbered consecutively, starting from zero, for improved compatibility with igraph. The indispensable components in the workflow are the Rshells compute_eigenvector_centrality, which calculate the eigenvector centrality using the igraph evcent() function. In this instance, directionality is ignored by specifying ‘directed = FALSE’. The output of the Rshells are the original arXiv paper identifiers for the *N* papers with the highest eigenvector centrality in each network. Beanshell components are then used to create a query and fetch the abstract page of these papers directly from arXiv using the Taverna Get_Web_Page_from_URL service. Embedded in the abstract extraction services are also XPath components that extract the abstract texts from the HTML files. The corpora are then passed to a pair of Rshells for drawing wordclouds on common words in these two extreme sets of abstracts using the tm and wordcloud R packages. The output of the workflow shows the word clouds for the *N* = 100 most central papers in the cit-HepPh (Fig. 5b) and cit-HepTh (Fig. 5c) citation networks. The phenomenology word cloud includes physical units, such as TeV, and experimental facilities such as the LHC particle accelerator. The theory word cloud, perhaps unsurprisingly, is dominated by “string”, “theory”, and the related terms “M-theory”, “supersymmetry”, “elevendimensional”, and so on. Using citation analysis and comparing measures of centrality in two citation networks distills the essential difference between two closely related fields—here two aspects of high-energy physics. Units of measurements have previously been shown to have the weakest co-occurrence coupling with terms such as “theory”, “model” and “simulation” in the field of analytical chemistry (Palmblad and Waaijer 2015).

### Connecting to Web Services and external databases

As shown in the example above, Taverna workbench can automatically analyze or generate networks directly from online data. Taverna can also invoke Web Services Description Language (WSDL) style Web services given the URL of the service’s WSDL document. The WSDL is an XML-based interface description language often used together with a Simple Object Access protocol (SOAP) to access the functions and parameters of a service. Many bibliographic resources are available through Web services, such as Web of Science (WoS) or PubMed Central (PMC). Some services, including the WoS, require authentication. An entire bibliometric study can be contained inside a single Taverna workflow that authenticates the user, if needed, takes the user queries, or questions of the study, generates the Web service requests, executes these, retrieves the data and proceeds with further (local) statistical analysis and visualization.

**Fig. 6:**
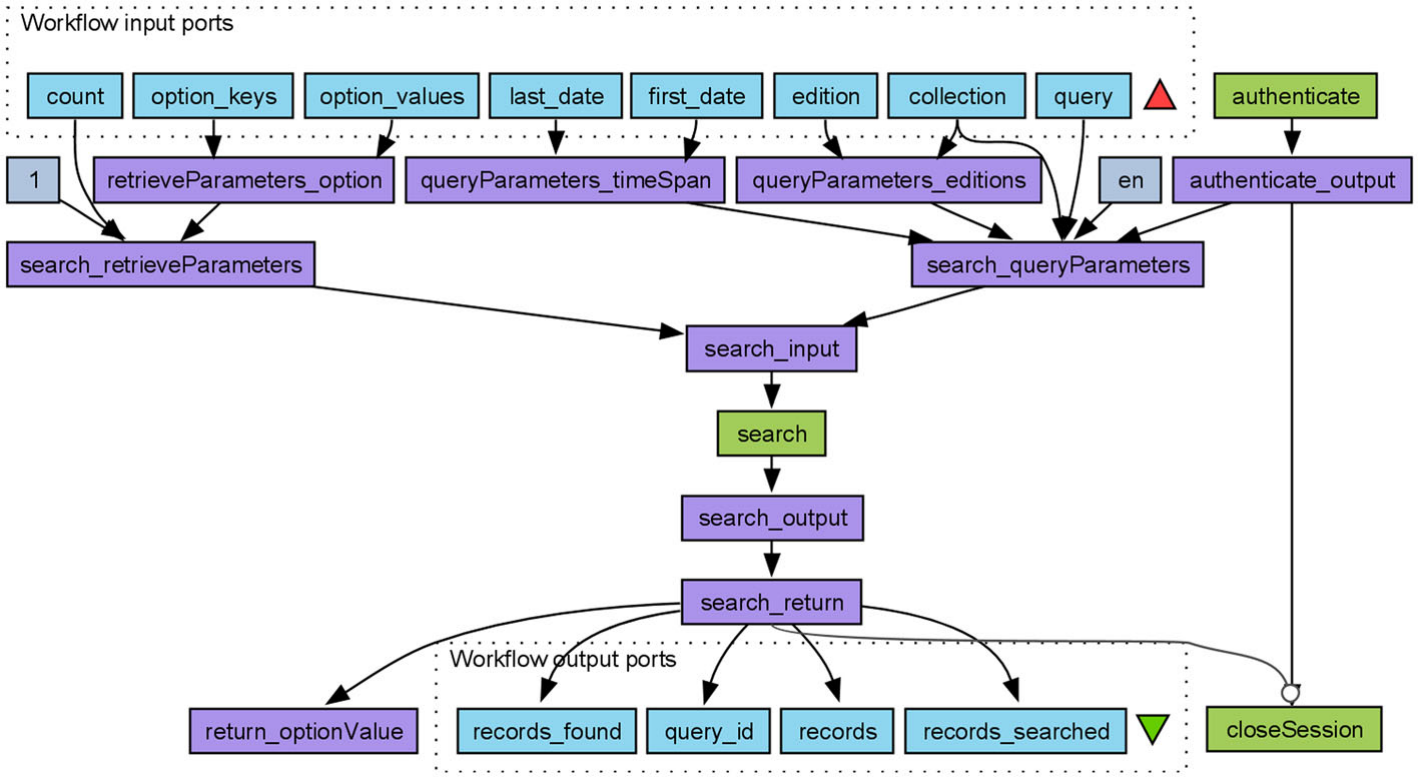
Taverna interface to the Thomson Reuters Web of Science Web services lite. This Web service has a relatively complex WSDL interface and also requires authentication. Taverna reveals the WDSL interface, allowing the user to understand what is required by, and what can be retrieved from, the service. The port names are the same as in the Thomson Reuters Web service documentation

**Fig. 7:**
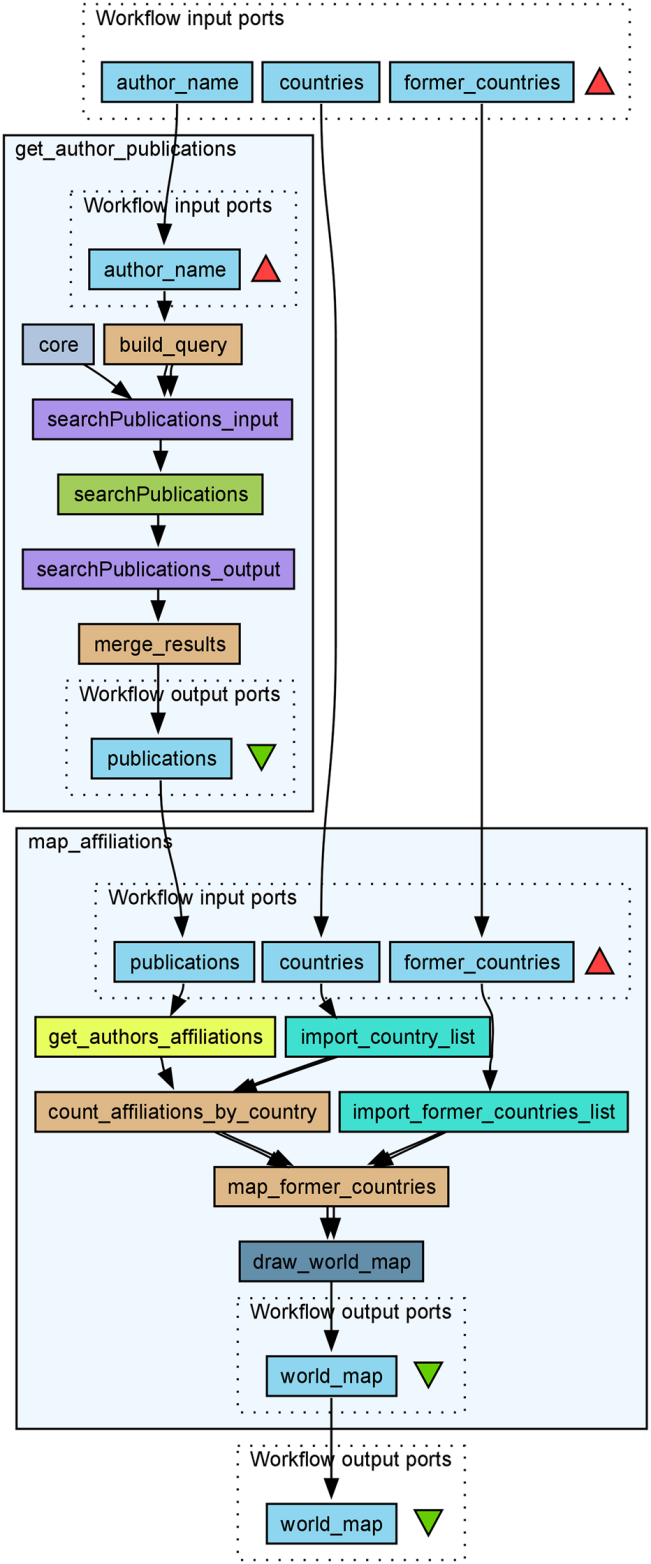
A simple workflow demonstrating retrieving bibliometric data using the EBI Europe PubMed Central WSDL Web service (*wild willow green*). Taverna handles the interface to the Web service, creating the SOAP request and retrieving selected results (*medium purple*). The output from this workflow is a world map showing the geographic distribution of collaborators. Alternatively, a time-lapse movie can be created using the “animation” package in R to show how the collaborations change over time

A Taverna workflow that invokes WSDL services from WoS to automatically execute a query may look like in Fig. 6. This Taverna workflow takes as input common search parameters and a generic WoS query string, and pass these to the Web service via the WoS WSDL interface. Values that have only one possible value, such as the language (English, “en”) are here hard-coded in the workflow as *Text constants*. A workflow that connects to the EBI Europe PubMed Central (PMC) SOAP Web service and maps the author affiliations article by article, ordered by publication year, is part of the workflow shown in Fig. 7 below. The output of the entire workflow is a world map showing the geographic trends collaborative patterns of an individual researcher (more on this below). The workflow can easily be adapted to show geographic trends in research topics, publications in a particular journal etc. All that needs to be modified are the PMC search query and the XPaths, and this can be done in a few mouse clicks without typing any code.

### Geographic analysis of publications

**Fig. 8:**
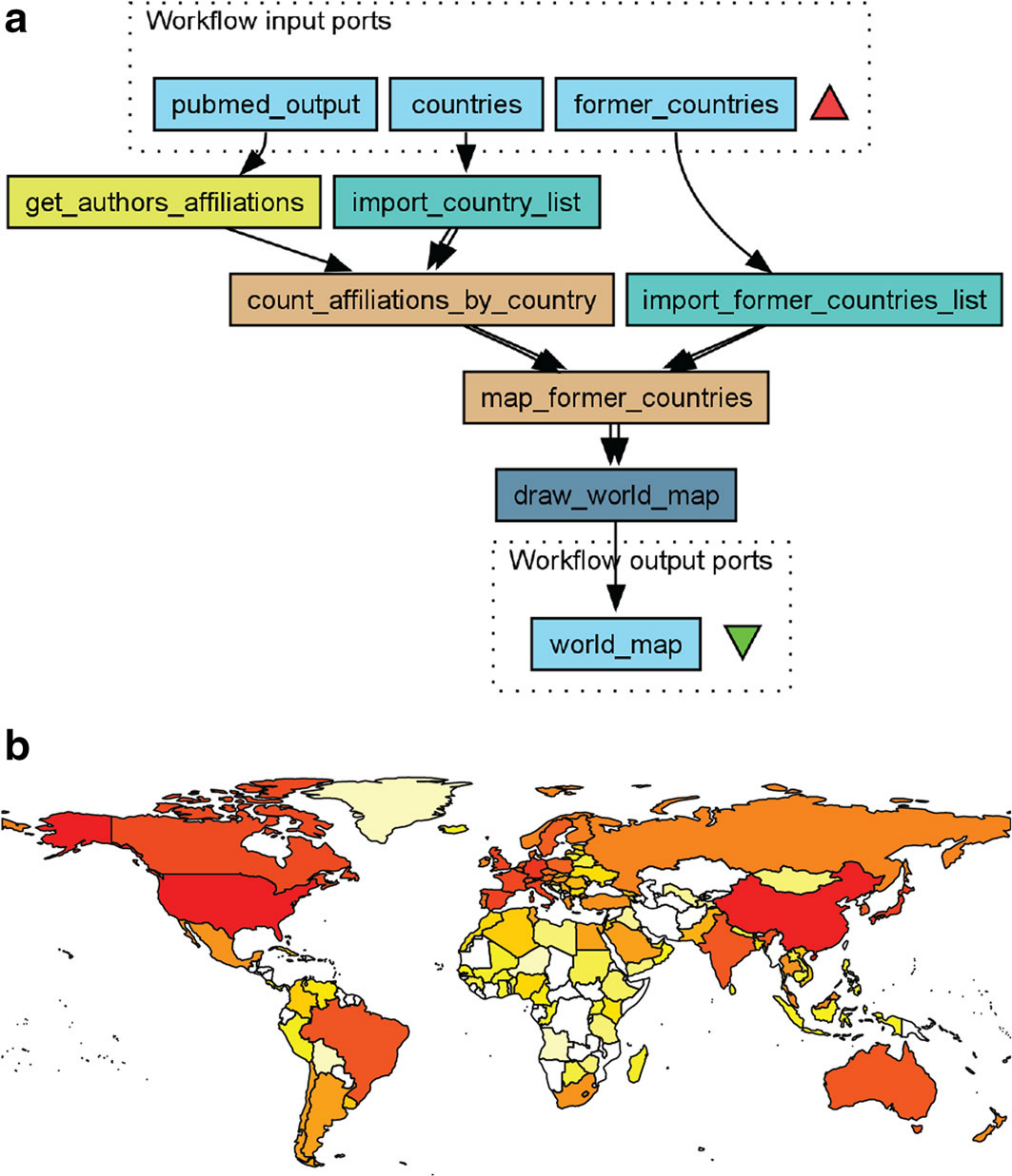
**a** PubMed XML output contains information on author affiliations as provided by the authors themselves. This Taverna workflow extracts extracting geographic information (here countries) and converts it to a standardized format (ISO 3166) from the PubMed XML output. The workflow counts the number of appearances of each country in the author affiliations in the XML file and uses the R package “rworldmap” to visualize them. rworldmap and similar tools require country names to be in a standard format, e.g. the three letter code from ISO 3166. The text mining component is therefore necessary to connect PubMed with geographic visualization. **b** Output of the workflow for the search string “(mass spectrometry[Title/Abstract]) AND (“2010/01/01”[Date-Publication]: “2014/12/31”[Date-Publication])” in PubMed, showing the geographic distribution of active (and actively publishing) researchers in the field of mass spectrometry in the past 5 years

Using the rworldmap package described above, we constructed another simple example workflow, *Compare_pubmed_results_geographically*, to project author affiliations onto a map of the world, displaying the number of publications on a particular topic per country (Fig. 8a). This example highlights how geographical (country) information can be extracted from the affiliation field in PubMed XML, matched to present-day countries in the ISO 3166-1 standard while transferring data from former countries (as defined in ISO 3166-3) to their successor states. This process works relatively well for publications later than ca. 1949, after we provided the workflow with a table linking former countries with their contemporary counterparts. The latter will obviously never be a perfect process, and some arbitrariness is unavoidable. For example, should research output from the former USSR be shared equally (on the map) between all fifteen independent states that emerged after the dissolution of the Soviet Union, or exclusively to the Russian Federation? Should it depend on where the authors were located at the time? Some borders, such as that between the former West and East Germany, have disappeared from the map in rworldmap. However, for visualization of research activity in the past two decades, rworldmap does the job well. rworldmap also allows some control of granularity and what area of the globe to plot. For example, Antarctica and small islands can be omitted without appreciable loss of accuracy. There are currently no human inhabitants on the Bikar Atoll in the Pacific, let alone research institutes.

The workflow in Fig. 8a takes a PubMed XML, extracts all author affiliations and maps these to present-day countries in ISO 3166-1, tallies the publications and maps the total number per country onto a current map of the world. This workflow is also available on myExperiment (Goble et al. 2010, http://myexperiment.org/workflows/4648.html). The results from running this workflow on the topic defined as all articles matching “mass spectrometry” in their title or abstract published between 2010 and 2015 is shown in Fig. 8b. As an alternative to starting from a PubMed XML file, we can connect the output from the PMC Web service as input to *Compare_pubmed_results_geographically* (Fig. 7). This combined workflow is also available on myExperiment. In addition to producing static maps, it is also possible to export a series of author affiliation maps as a movie using the “animation” R package.

**Fig. 9:**
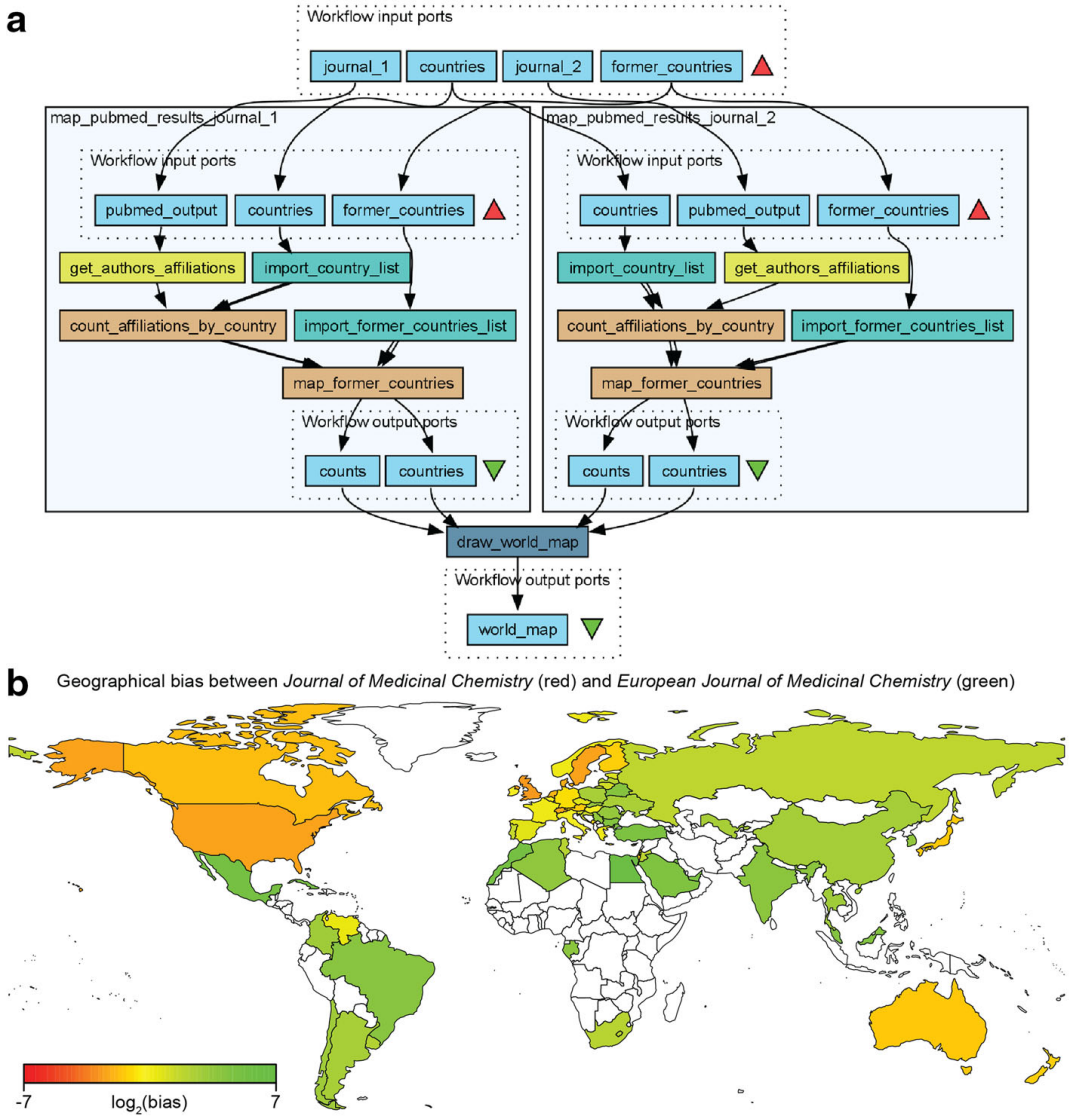
**a** A workflow *Compare_two_journals_geographically* reusing the embedded *map_affiliations* workflow matching author affiliations with countries in Fig. 6 for analyzing geographical bias in two medicinal chemistry journals: the American Chemical Society (ACS)-published *Journal of Medicinal Chemistry* and the *European Journal of Medicinal Chemistry* between 2000 and 2015. **b** The results of the workflow above with publication bias shown as *color* from *red* to *green* representing a bias of a factor 2^7^ = 128 in publishing in the ACS over the European journal. The numbers of publications were normalized to the total number of articles in the two journals (11,219 articles in *Journal of Medicinal Chemistry* and 5842 articles in the *European Journal of Medicinal Chemistry* respectively). The most recent Thomson Reuters journal impact factor is 5.447 for the ACS journal (2014) and 3.432 for the European journal (2013) respectively

Journals covering in the same scientific field may have regional bias, with for example researchers based in the US preferentially publishing in an American journal and European researchers preferring a European journal. To investigate whether there is such a bias in the field of medicinal chemistry, we looked specifically at the *Journal of Medicinal Chemistry* (published by the American Chemical Society) and the *European Journal of Medicinal Chemistry*. To this end we assembled the workflow shown in Fig. 9a. This workflow analyzes the geographical bias in author affiliation between any two journals, not just the two investigated here. The output is again a map generated by rworldmap, this time with a color gradient representing the relative number of publications in the two journals for each country (Fig. 9b). It is clear from this analysis that authors from many Western European countries have a preference for the American journal. This may have something to do with this journal having a higher journal impact factor (as measured by the Thomson Reuters journal impact factor) and consequently being considered more prestigious in the field. On the other hand, other Western European countries such as France and Italy do not show this preference. This may be explained by the fact that a sizeable share of the editorial board is comprised of researchers working in France or Italy.

## Discussion and conclusions

The use of scientific workflows in bibliometrics is still in its infancy. The direct support of R inside Taverna workflows is particularly useful for bibliometrics and scientometrics. A number of R packages for bibliometric analysis have recently been released, ranging from simple data parsers such as the “bibtex” package (Francois 2014) for reading BibTeX files to libraries or collections of functions for scientometrics, such as the *CITAN* package (Gagolewski 2011). The latter package contains tools to pre-process data from several sources, including Elsevier’s Scopus, and a range of methods for advanced statistical analysis. The igraph package itself comes with some functions specifically for bibliometric analysis, e.g. “cocitation” and “bibcoupling”. Clustering or rearranging the graph spatially so that strongly connected words appear closer together is possible with igraph, but may also be assisted by other packages. We opted for showing a few simple but more or less representative examples here. Much more complex analyses can be designed based on or using the workflows and components here as a starting point. We did not include any advanced text mining functionality for homonym disambiguation or natural language processing. The “openNLP” R package currently in development provides an interface to openNLP (Hornik 2014) and may be used to extract noun phrases and refine the analyses.

In the examples here, we could show that individual language preferences can dominate when comparing two authors working in the same field. We could also show that the geographical bias between two medicinal chemistry journals, one European and one published by the American Chemical Society, probably has more to do with impact factor and perceived prestige than author location, based on the observation that researchers from the European countries usually ranking high in international research surveys, i.e. Denmark, the Netherlands, Sweden, Switzerland and the United Kingdom, also have the strongest preference for publishing in the higher-impact factor American journal. To the extent that such rankings are based on impact factors, this is of course in part a circular argument. We also observe that European countries well represented on the editorial board of the European journal, e.g. France and Italy, show no preference for the American journal. This is probably not a coincidence.

Scientific workflow managers are powerful tools for managing bibliometric analyses, allowing complete integration of online databases, Web services, XML parsers, statistical analysis and visualization. Workflow managers such as Taverna eliminate manual steps in analysis pipelines and provide reusability and repeatability of bibliometrics analyses. All workflows for bibliometrics and scientometrics presented here can be found in the myExperiment group for Bibliometrics and Scientometrics (http://myexperiment.org/groups/1278.html).
